# Pulmonary aspiration in preschool children with cystic fibrosis

**DOI:** 10.1186/s12931-018-0954-1

**Published:** 2018-12-17

**Authors:** D. Clarke, I. Gorman, F. Ringholz, M. McDermott, DW. Cox, P. Greally, B. Linnane, P. Mc Nally

**Affiliations:** 1 National Children’s Research Centre, Crumlin, Dublin 12, Ireland; 2 Our Lady’s Children’s Hospital, Crumlin, Dublin 12, Ireland; 3 National Children’s Hospital, Tallaght, Dublin, Ireland; 4 Department of Paediatrics, Royal College of Surgeons in Ireland, Dublin, Ireland; 5 University Hospital Limerick, Limerick, Ireland; 6 Graduate Entry Medical School and Centre for Interventions in Infection, Inflammation & Immunity (4i), University of Limerick, Limerick, Ireland

## Abstract

Pulmonary aspiration of gastric refluxate (PAGR) has been demonstrated in association with pulmonary inflammation in school aged children with Cystic Fibrosis (CF). We sought to determine if similar findings were present in preschool children. Pepsin was measured in Broncho-alveolar lavage (BAL) fluid collected from clinically stable preschool children with CF and controls. Elevated pepsin levels were found in a subgroup of children with CF, but this was not found to be associated with pulmonary infection, pulmonary inflammation or respiratory or gastrointestinal symptoms.

## Introduction

Gastro-oesophageal reflux (GOR) is the involuntary passage of gastric content into the oesophagus. Gastro-oesophageal reflux disease (GORD) is present when GOR causes adverse consequences such as pain, failure to thrive or pulmonary aspiration. The reported prevalence of GORD in CF is between 35 and 81% and has been associated with reduced lung function and increased cough in older patients [1, 2]. We have previously demonstrated that PAGR occurs in a subgroup of school-aged children with CF and is associated with more pronounced airway inflammation [3].

There has been much focus recently on the detection of evolving lung disease in preschool children with CF, where traditional outcome measures are poor and children can be largely asymptomatic despite the development of irreversible structural lung disease [4, 5]. The focus of pulmonary management in CF is increasingly moving towards aggressive prevention of disease, where possible, rather than solely targeting clinically evident complications. Pulmonary aspiration can cause significant lung disease [6] but is difficult to diagnose clinically [7]. We sought to discover if pulmonary aspiration was occurring in a group of clinically stable preschool children with CF and, if present, was associated with greater airway inflammation.

## Methods

Clinically stable preschool children with CF undergoing routine surveillance bronchoscopy were recruited through the Study of Host Immunity and Early Lung Disease in Cystic Fibrosis (SHIELD CF). Clinical data were prospectively collected on all children at CF clinic with a standardised clinical information sheet. Non-CF controls were children without CF undergoing bronchoscopy for clinical reasons. Informed parental consent was obtained and the study had ethical approval from the institutional review board of Our Lady’s Children’s Hospital, Crumlin, Dublin.

Bronchoscopy was performed under general anaesthesia via laryngeal mask airway. Bronchoalveolar lavage (BAL) samples were obtained by instilling two sequential aliquots of 1 ml/kg of sterile normal saline per lobe, into the right middle lobe and lingula (or other affected lobe). Retrieved samples were then pooled. Differential cell counts were performed microscopically after staining with Haematoxylin and Eosin. Pepsin concentrations were measured in control and CF BAL using a commercial human pepsin ELISA (USCN Life Science Inc. China). Interleukin 8 (IL-8) was measured in CF BAL by ELISA as previously described [3]. Neutrophil Elastase (NE) activity of CF BAL samples was quantified using the substrate N- (Methoxysuccinyl)-Ala-Ala-Pro-Val p-nitroanilide as previously described [8]. Differences in normally distributed data were analysed by Students t-test, chi squared or Pearson, and non-normally distributed data with Mann Whitney and Spearman. Significance is denoted as a *p* value < 0.05.

## Results

**Fig. 1:**
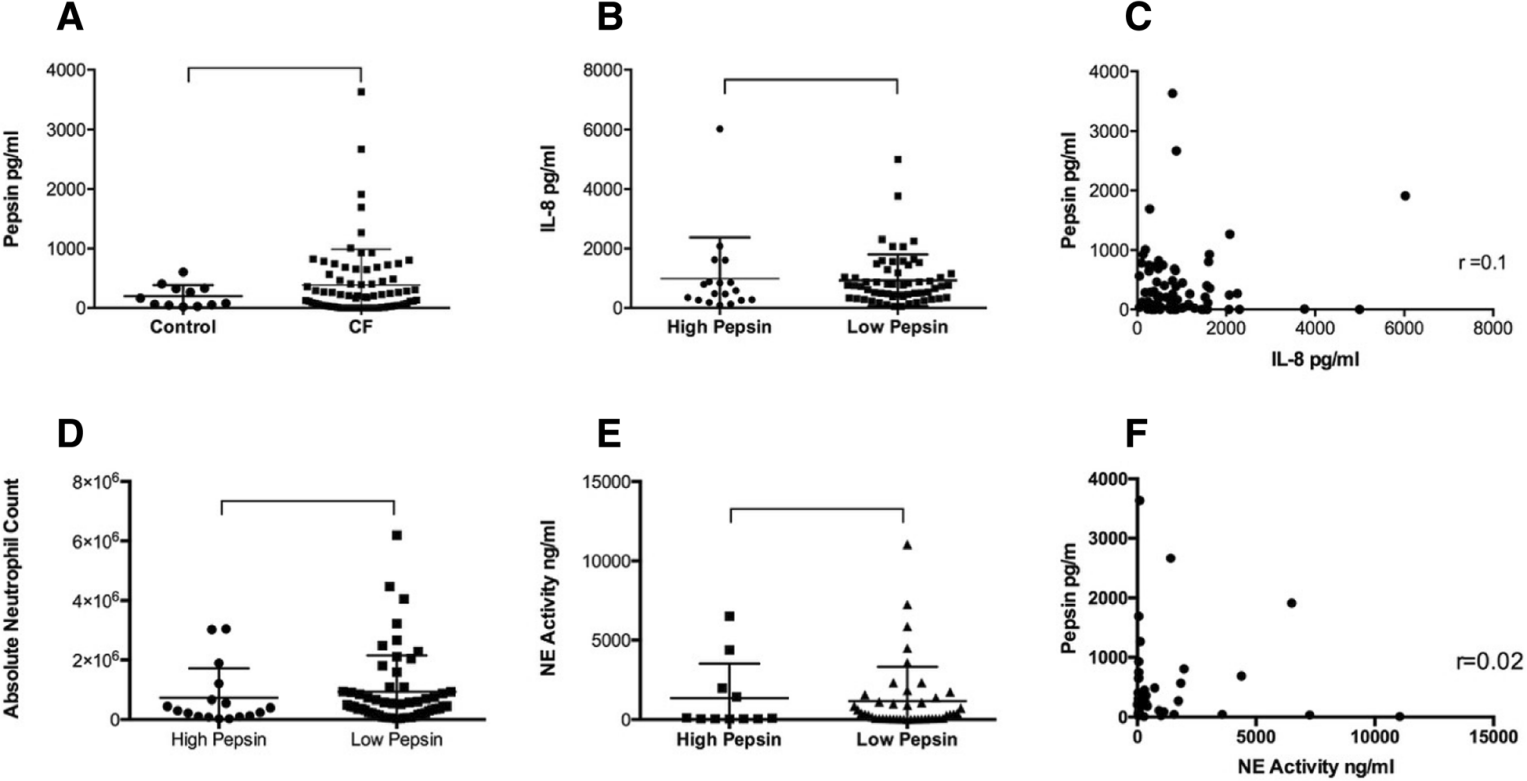
**a** BAL levels of Pepsin among children with CF and controls (*p* value 0.81), **b** IL-8 levels in BAL among those with CF in the high pepsin and low pepsin groups (*p* value 0.49), **c** Correlation between BAL pepsin and BAL IL-8 in children with CF, **d** Absolute neutrophil count in BAL among children with CF with high and low pepsin levels (*p* value 0.31), **e** Neutrophil elastase (NE) activity in BAL among children with CF with high and low pepsin levels (*p* value 0.98), **f** Correlation between BAL pepsin and BAL NE activity in children with CF

Eighty nine children were included in the study, 77 with CF [mean age 3.7 years (range 0.86–6.4)] and 12 Control [3.6 years (1.5–7.8)] (Table 1). No patients had more than one BAL. BAL pepsin levels were not significantly different between the groups (385 ± 68.7 pg/ml v. 198 ± 54.2 pg/ml [mean ± SEM]) (Fig. 1a). A subgroup of children with CF (18/77 [23%]) had particularly high levels (high pepsin, > 2 SD above control mean [> 573 pg/ml – normally distributed data]) compared to all others with CF (low pepsin). There was no difference in levels of IL-8, BAL neutrophil counts, proportion with free NE in BAL or NE levels (where detected) between the high and low pepsin groups (Fig. 1b, d, e). No associations were found between BAL levels of pepsin and either IL-8 or NE (where detected) (Fig. 1c, f). Recognised CF pathogens (*S. aureus, H. influenza, P. aeruginosa, S. maltophilia*) were found in BAL from 41 of 77(53%) children with CF. There was no statistically significant difference in the presence of these organisms, either individually or collectively between the high and low pepsin groups. There was a low prevalence of *P. aeruginosa* (3.9%) and MRSA (1.3%) overall within the CF group. There was no significant difference in any of the gastrointestinal (3/18 and 5/59 with GOR symptoms [*p* = 0.136]) or respiratory symptoms related to pulmonary aspiration or GOR between children with CF with high and low pepsin levels.

**Table 1:** Baseline demographics for the study populations

	CF (*n* = 77)	Controls (*n* = 12)	*p* value
Age, mean in years (range)	3.75 (0.86–6.4)	3.6 (1.5–7.8)	0.747
Gender	26F/51M	3F/9M	0.547
BMI, mean (range)	16.6 (15–20.6)	17.6 (16.1–19.7)	0.156
Genotype			
F508del Homozygotes	50	N/A	N/A
F508del Heterozygotes	17	N/A	N/A
Other	10	N/A	N/A

## Discussion

We demonstrate in this study that almost 1 in 4 preschool children with CF have elevated levels of BAL pepsin, suggesting sub-clinical PAGR. In contrast to the findings of our previous study in older children with CF [3], in this study there is no evidence that the presence of pepsin in BAL was associated with pulmonary inflammation. We have little understanding of the natural history of PAGR in children with CF, particularly as it relates to frequency of occurrence and variability over time. PAGR may need to be present for a number of years before it is associated with recognisable pulmonary inflammation. This will require further study.

We specifically prospectively collected clinical data on respiratory and gastrointestinal symptoms (heartburn, vomiting, post-tussive vomiting, coughing pattern) to attempt to determine if there was a recognisable clinical phenotype associated with PAGR. We found no difference in symptoms in those with high pepsin levels compared to the rest of the CF group. The diagnosis and treatment of PAGR is a challenging area. There is likely a significant overlap between the symptoms of pulmonary aspiration and typical lung disease in CF, and while it is possible to measure GOR, and to radiologically assess pulmonary aspiration during swallowing, we lack a specific and sensitive commercially available diagnostic test for the aspiration of refluxate, the key biological event of concern. Because we have no available treatment to prevent aspiration of material that refluxes into the oesophagus or pharynx, treatment for PAGR involves prevention of GOR, which may ultimately entail fundoplication, not an insignificant undertaking. Proton pump inhibitors, commonly used in CF, may well worsen the situation [9, 10].

While it is positive that neither changes in pulmonary pathogens or pulmonary inflammation were found in those with PAGR, we have nonetheless discovered a potentially significant biological abnormality that occurs in a subset of patients. Unfortunately, a perfect biomarker for pulmonary aspiration does not exist. Longitudinal BAL pepsin measurements, perhaps in combination with other previously published PAGR biomarkers such as bile acids [2], with robust medium term outcome measures, will be required to determine if early PAGR is associated with subsequent clinically relevant adverse outcomes, an approach we plan with this cohort.

## Abbreviations

BAL: Broncho-alveolar lavage
CF: Cystic fibrosis
ELISA: Enzyme linked immunosorbent assay
GOR: Gastro-oesophageal reflux
GORD: Gastro-oesophageal reflux disease
IL-8: Interleukin 8
NE: Neutrophil elastase
PAGR: Pulmonary aspiration of gastric refluxate
SHIELD CF: Study of host immunity and early lung disease in cystic fibrosis
